# A life‐devastating cause of gastroenteritis in an immunocompetent host: was it suspected?

**DOI:** 10.1002/ccr3.1338

**Published:** 2017-12-15

**Authors:** Ahsan Wahab, Siddique Chaudhary, Ali Ahmad, Vidya Kollu, Susan Smith

**Affiliations:** ^1^ McLaren Regional Medical Center Flint Michigan

**Keywords:** Bacteremia, death, gastroenteritis, immunocompetent, listeriosis

## Abstract

A 44‐year‐old immunocompetent man with gastroenteritis received supportive care and empiric antibiotics. After an initial response, he developed septic shock. Blood cultures grew Gram‐positive bacilli with antibiotic adjustment. However, he succumbed within 36 h. After the patient's death, *Listeria monocytogenes* was identified on blood culture.

## Introduction

According to one estimate, there are 9.4 million foodborne infections annually acquired in the United States ranging from self‐limited gastroenteritis (GE) to severe systemic illness 1. Among them, bacteria are responsible for 3.6 million infections, causing hospitalizations and deaths in more than 60% of cases 1. Since the first outbreak in 1981*, Listeria monocytogenes*, a Gram‐positive bacillus, has been implicated in both outbreaks and sporadic cases. During 2009–2011, 1651 patients were reported with invasive listeriosis (IL); of those 21% died representing an extremely high case‐fatality rate 2. Although not among the most common causes of hospitalizations for foodborne illnesses, *Listeria* is responsible for 19% of those leading to death, making it the third leading cause of foodborne‐related mortality 1. A worldwide literature review reveals that 55–77% of nonperinatal cases occur in males 3.

*Listeria monocytogenes*, an intracellular pathogen, ubiquitously occurs in nature and can be found in both animal products (unpasteurized milk, cheese, undercooked meat, seafood, etc.) and nonanimal sources such as vegetables, soil, and water. *L. monocytogenes* is thought to be an underrecognized cause of self‐limited GE because it is not detected on routine stool culture, requiring special media for growth. It tends to cause invasive illness in extremes of age, pregnancy, and immunocompromised (IC) states (HIV, malignancy, chemotherapy, radiation therapy, steroids, diabetes, cirrhosis, renal failure, and alcoholism) 2.

The elderly are four times more likely to acquire invasive illness than the nongeriatric population, the latter being at greatest risk when IC. In contrast, immunocompetent hosts rarely present with invasive disease due to their intact cellular immunity. Although the exact mechanism remains unclear, a few authors have speculated that the size of the bacterial inoculum may overwhelm intact immunity at times leading to severe systemic illness in the immunocompetent 4, 5, 6.

## Case Report

A 44‐year‐old African American man with a prior psychiatric history came to the emergency department with diarrhea, lethargy, and decreased oral intake for 4–5 days. He reported multiple bouts of vomiting and nonbloody loose stools. He reported no consumption of unpasteurized milk or undercooked meat and had no recent travel. He abused cocaine and cannabinoids but denied current or previous intravenous (IV) drug use. He lived alone and reported no ill contacts. His history was negative for multiple or same‐sex partners. Medications included psychotropics (olanzapine and bupropion) and antihypertensives. He reported subjective fever but no headache, photophobia, neck stiffness, or focal weakness. Vitals showed hypotension (99/65 mmHg), tachycardia (heart rate of 140/min), and fever (100.6°F). He was saturating well on room air without distress. On physical examination, he appeared ill and lethargic. He was arousable and oriented times three. Cranial nerves were intact. Signs of meningeal irritation (nuchal rigidity, Kernig's and Brudzinski's signs) were absent, and there was no motor or sensory deficit. Oral mucosa was dry, and sclera was jaundiced. Respiration was nonlabored. Lungs were clear to auscultation. Heart rhythm was regular; S1 and S2 were normal without murmur. Bowel sounds were present. The abdomen was soft without distention. There was mild tenderness in the periumbilical area and the left lower quadrant. Musculoskeletal and skin exams were unremarkable except for decreased skin turgor. Complete blood count showed leukocytosis with neutrophilia, lymphopenia, and thrombocytopenia. Metabolic panel revealed acute kidney and liver injury and metabolic acidosis. Lactic acid was normal on admission, and hepatitis screen was negative (See Table 1 for values). CT of abdomen and pelvis was performed, which revealed enteritis and colitis of the descending sigmoid colon along with a small pelvic effusion.

**Table 1:** Laboratories of the patient with *Listeria monocytogenes* septicemia^a^

Labs	At Admission	After 24 h
Hemoglobin (13.5–17.7 g/dL)	12.0	9.5
Hematocrit (39.8–52.3%)	35.5	28.9
Mean corpuscular volume (80.0–94.0 fL)	79.2	78.1
Mean corpuscular hemoglobin concentration (33.0–37.0%)	33.8	32.9
Red blood cell count (4.70–6.10 × 10^6^/*μ*L)	4.48	3.70
White blood cell count (4.50–11.00 × 10^3^/*μ*L)	13.65	0.79
Absolute Neutrophils (1.40–6.50 × 10^3^/*μ*L)	12.69 (89%)	0.60 (75.9%)
Absolute Lymphocytes (1.20–3.40 × 10^3^/*μ*L)	6.8 (5%)	0.14 (17.7%)
Platelet Count (140–440 × 10^3^/*μ*L)	109	50
Glucose (70–105 mg/dL)	149	73
Blood urea nitrogen (7–22 mg/dL)	15	12
Creatinine (0.50–1.50 mg/dL)	2.05	1.58
GFR for African American (60–240 mL/min)	43	58
Calcium (8.5–10.5 mg/dL)	8.1	6.3
Sodium (134–145 mmol/L)	130	132
Potassium (3.5–5.1 mmol/L)	3.8	3.7
Chloride (98–112 mmol/L)	89	99
Carbon Dioxide (24–30 mmol/L)	24	12
Anion Gap (3–15 mmol/L)	17	18
Albumin (3.5–5.0 g/dL)	2.5	1.6
Globulin (2.1–3.7 g/dL)	3.8	2.8
Albumin/Globulin ratio (1.1–2.2)	0.7	0.6
Bilirubin total (0.2–1.3 mg/dL)	9.2	8.1
Alanine aminotransferase (8–40 U/L)	326	787
Alkaline phosphatase (39–117 U/L)	123	59
Aspartate aminotransferase (7–56 U/L)	253	256
Lactate (0.5–2.0 mmol/L)	1.9	3.2, 5.6, 9.1, 11.4

a Normal range of values shown in parenthesis.

**Figure 1:**
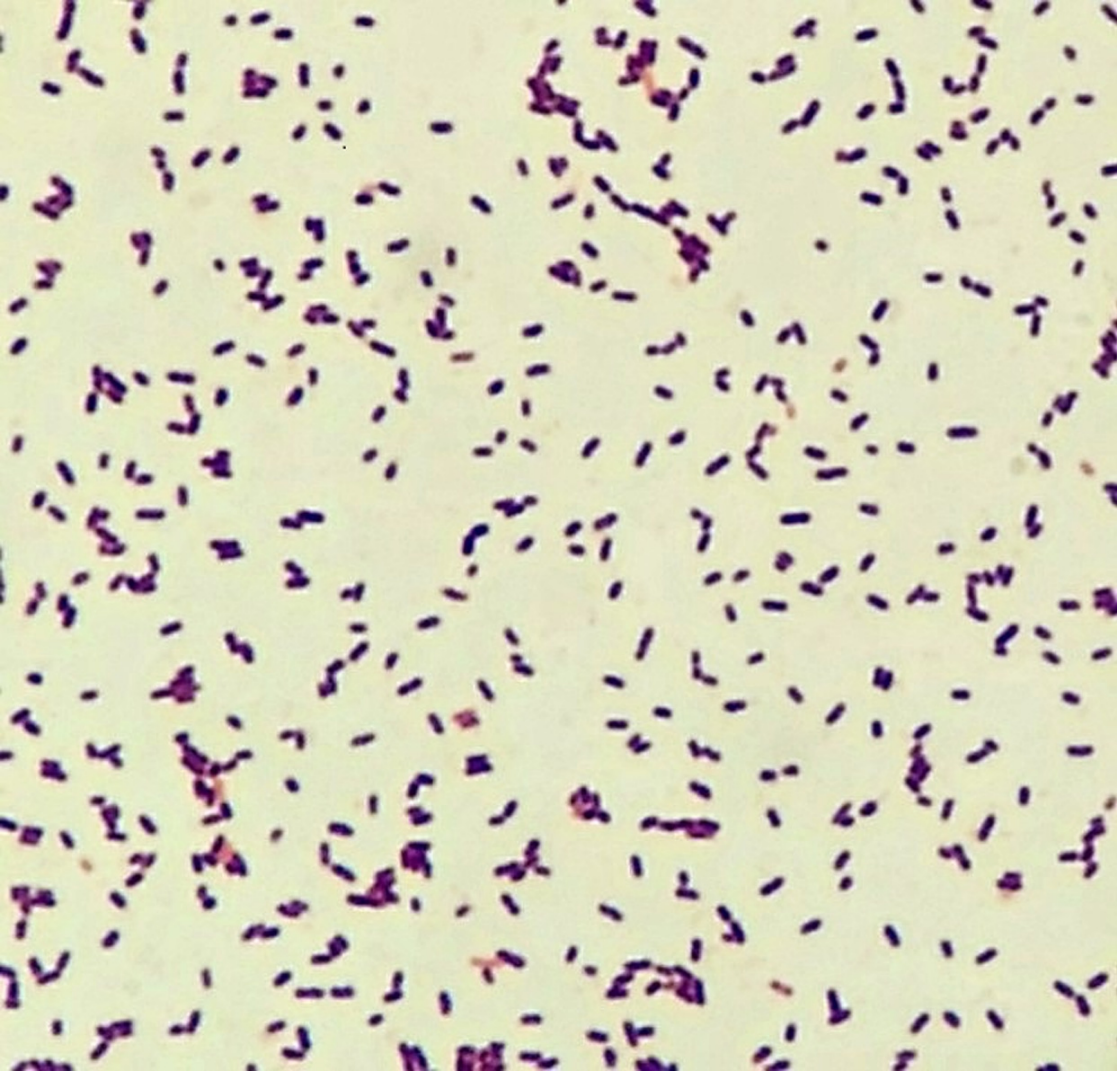
Microscopic examination of *Listeria monocytogenes* with Gram stain of blood culture.

Blood cultures (BC) were drawn before administering empiric therapy for common pathogens, that is, one gram of ceftriaxone and 500 mg of metronidazole intravenously within the first hour of arrival. He also received aggressive fluid resuscitation at 30 mL/kg of ideal body weight within 3 h of admission. Over a few hours, his vital signs normalized. After initial antibiotics, the patient was placed on one gram of IV ceftriaxone daily and 500 mg of IV metronidazole three times daily in addition to maintenance fluid replacement with normal saline at a rate of 125 mL/h. Stool specimen was sent for Gram stain, culture, and *Clostridium difficile* toxin. In the absence of a clinical picture of meningoencephalitis, lumbar puncture was not performed. Cerebral CT was normal. In spite of an initial response, hypotension recurred and plateaued, and tachycardia persisted, pulse ranging 120–130 beats/min though the patient had received almost 6–8 L of fluids within 12 h. Nevertheless, the patient showed continuing deterioration requiring intubation and pressor support for multiorgan failure. By the second day, BC grew Gram‐positive bacilli with identification pending. Ceftriaxone was stopped, and the patient was started on IV piperacillin‐tazobactam (3.375 mg every eight hourly), IV vancomycin with pharmacy‐dosing and oral vancomycin (250 mg four times a day, for suspected severe *C. difficile* colitis) in addition to metronidazole. His repeat complete blood count showed severe leukopenia and thrombocytopenia (Table 1). Due to refractory septic shock, the patient developed progressive lactic acidosis and required multiple vasopressors (norepinephrine, epinephrine, and dopamine were added in a sequential manner). Stool workup, including *C. difficile* toxin and cultures, was negative with only a few WBCs on microscopy. HIV screen was negative. The patient continued to deteriorate and succumbed within 36 h of admission despite aggressive resuscitative treatment and antibiotics. BC was reported positive for *L. monocytogenes* after 48 h (Fig. 1)**,** 12 h after the patient's death. Retrospectively, this pathogen was not suspected in our patient given his intact cellular immunity and the lack of suspicious food exposure. Precipitous deterioration led to his demise before identification of the pathogen could allow targeted treatment.

## Discussion

Invasive listeriosis is defined as a severe bacterial infection caused by *L. monocytogenes* with subsequent retrieval or isolation of the organism from sites that are normally sterile, including blood, cerebrospinal fluid (CSF), or products of conception. Generally, febrile GE, a noninvasive illness, precedes the development of IL. Among individuals with a deficient cellular immunity, such GE could be overwhelming, leading to systemic complications. Like other foodborne infections, *Listeria* GE has a short incubation period (IP), with 50% of cases developing within 24 h of exposure. In contrast, IL (bacteremia, meningoencephalitis, and pregnancy‐associated cases) has an extended IP (Median IP: 8 days, range: 1–67 days), with bacteremia having the shortest IP (Median IP: 2 days) 7.

To date, in foodborne illnesses, serotypes 4b, 1/2a, and 1/2b have been implicated in more than 90% of cases, with serotype 1/2a most common in bacteremia cases 8. Multiple factors account for the virulence of *Listeria* and the immune response of hosts. After ingestion, *Listeria* crosses the intestinal epithelial cell membranes via transcytosis, invading the mesenteric lymph nodes to gain circulatory access. Protein internalin mediates the initial cell entry of *Listeria* by binding to the E‐cadherin receptors present on host cells. Listeriolysin O, a secreted hemolytic protein, acts on cholesterol‐containing membranes and helps the organism escape phagocytic vacuolization 8.

*Listeria* GE, in general, is a self‐resolving illness presenting with fever, nausea, vomiting, headache, watery diarrhea, and arthromyalgia 4. Bloody diarrhea, though uncommon, if present, could be the sign of severe illness. According to one study, *Listeria* is not a common cause of foodborne‐related hospitalizations but demonstrates a relatively severe course compared with other foodborne pathogens 9. In contrast to self‐limited GE, there is an ominous disease caused by *Listeria* with systemic dissemination, usually in immunodeficient hosts, presenting as septicemia with or without neuroinvasion 10. In addition to immune dysfunction, host factors altering the intestinal milieu, such as medications and intercurrent or concurrent intestinal diseases, facilitate invasiveness leading to IL. Bacteremia can lead to other forms of IL including central nervous system infections and perinatal disease. Therefore, in the context of recent febrile GE, signs of these illnesses should be interpreted with a high index of suspicion.

Like other causes of bacteremia, listeriosis can cause fulminant disease, presenting with severe systemic illness leading to distributive shock and multiorgan failure, usually in the immunocompromised. Although there are several case reports of *Listeria* meningoencephalitis in immunocompetent hosts, the presence of sepsis secondary to *Listeria* in otherwise healthy individuals, as in our patient, is extremely uncommon 3, 11, 12, 13, 14, 15. Only the two patients reported by Von Rotz et al. involved otherwise healthy individuals in sporadic IL. They illustrated two immunocompetent individuals (a 16‐year‐old son and 46‐year‐old father) with GE and bacteremia after they consumed a suspected food source (ham and cheese sandwiches during camping). Rapid identification *of Listeria* was achieved by matrix‐assisted laser desorption/ionization time‐of‐flight (MALDI‐TOF), a relatively new and not readily available test. Therefore, they were treated with first‐line antilisterial antibiotics resulting in recovery 16. In contrast to these cases, our patient (1) had no suspected food exposure; (2) was extremely sick upon arrival showing evidence of shock; (3) had signs of severe systemic failure including hepatic and renal injury; and (4) yet did not receive antilisterial antibiotics because this organism was not suspected. *L. monocytogenes* identification was not made until after his death; MALDI‐TOF was not readily available in this community.

The diagnosis of listeriosis demands a high clinical suspicion implying timely information regarding immunodeficiency and point exposure to a contaminated food source. However, extreme scenarios and nonspecific features in the absence of clinical clues add to diagnostic uncertainty and require particular attention. Blood and CSF cultures are vital to the diagnosis and should always be performed in invasive disease. Sophisticated testing of MALDI‐TOF mass spectrometry on initial blood culture growth can definitively identify organisms before conclusive culture results 16, 17.

Morphologically, *Listeria* can resemble corynebacteria or pneumococci on Gram stain, so interpretations should proceed cautiously particularly in IC patients. A stool culture is not helpful in invasive disease; in cases of suspected GE, special media are required. Polymerase chain reaction on CSF has been developed to detect *hly* gene (encodes Listeriolysin O antigen), but is not widely available; its diagnostic role in bacteremia has not been defined. Serum titers of antibodies against Listeriolysin O antigens can be positive and should be considered.

Antibiotics of choice in IL are ampicillin or penicillin G with or without aminoglycosides for synergism 10. Required duration of therapy is unclear but at least 2–4 weeks treatment in the immunocompetent and 3–8 weeks in IC individuals should be administered. Bacteremia with meningoencephalitis may require protracted treatment 10. Trimethoprim‐sulfamethoxazole is an alternative in patients with penicillin allergy, in cases refractory to initial treatment with or without ampicillin, or while switching IV to oral upon clinical improvement. Postantibiotic or postdischarge follow‐up is necessary for an early recognition of relapse and appropriate clinical management.

Cases of IL with isolated septicemia are quite rare and create diagnostic dilemmas when risk factors are absent or have not been reported. Moreover, as the antibiotic of choice is ampicillin or penicillin G in listeriosis, targeted treatment could be delayed because this is not usually considered first‐line therapy in acute, febrile GE. Hence, patients might deteriorate due to delayed treatment, nonadministration of antibiotics, or inappropriate choices for this organism as in our patient.

## Conclusion

We propose that if severe sepsis does not improve after initial empiric treatment for GE, physicians should have a low threshold for the initiation of ampicillin or penicillin G, even when risk factors for *Listeria* are absent or suspected exposure has not been reported.

## Consent

Informed consent was obtained from the patient's sister for the publication.

## Authorship

AW: took care of the patient and did the literature search. He wrote the preliminary manuscript and patient's case summary. SC: helped in writing the discussion part of the manuscript and made some corrections in the manuscript. AA: wrote the manuscript and coordinated the project. He provided the pathological images. VK: provided his specialized and technical help regarding the completion of the manuscript. SS: edited the whole manuscript at the end, checked the accuracy of references, and provided the critical review of the article.

## Conflict of Interest

All authors have no conflicts of interests.
